# Algorithmic hyperindividualisation and the transformation of vulnerability in substance use prevention

**DOI:** 10.3389/fdgth.2026.1956219

**Published:** 2026-10-02

**Authors:** Christoph Dockweiler, Carlotta Louisa Westfeld, Tessa Schulenkorf

**Affiliations:** Department of Social Sciences, Faculty of Arts and Humanities, University of Siegen, Siegen, Germany

**Keywords:** artificial intelligence, digital public health, health inequalities, personalisation, prevention paradox, substance use prevention, vulnerability

## Abstract

Generative artificial intelligence is entering substance use prevention through applications that tailor health information to the individual in real time. These systems promise to reach vulnerable groups more precisely than mass communication, and they rest on individual-level models of behaviour change. This article asks what happens to the concept of vulnerability once it becomes something such a system can read and act upon. It draws on the public health literature on vulnerability as a multilevel construct, the theory of fundamental causes, Rose’s prevention paradox, and critical work on algorithmic systems and precision public health. On this basis it develops the Vulnerability Transformation Hypothesis. The article first distinguishes hyperindividualisation from the tailoring it intensifies. What is new is the inference of undeclared attributes, continuous re-profiling, open-ended generation, and operation at near-zero marginal cost per message. The hypothesis is stated as three claims of decreasing certainty. A near-analytic addressability claim holds that a system addressing users as individuals has only individual-level levers. A substantive and conditional re-encoding claim states that, for any system presenting itself as a means of reaching the vulnerable, the operationalised concept of vulnerability is reconstructed into a constellation of individual attributes. Structural information that enters an individual model returns as a predictor of personal risk rather than as a target of action. This transformation is representational rather than ontological, since the structural causes persist even as they recede from what the system can represent and change. A contingent displacement claim adds that, at the level of the field rather than the single system, the success of individualised tools may displace structural effort, a tendency documented as lifestyle drift. The article separates its claim from the precision public health debate and offers four falsifiable predictions. Among them is a discursive test of whether systems that invoke vulnerability then present individual tailoring as the means of addressing it. Such a pattern would evidence substitution of the concept, not mere inconsistency. The article illustrates the coding scheme across contrasting systems, so that others can test and revise the hypothesis.

## 1 Introduction

Generative artificial intelligence is increasingly being explored for substance use prevention. A new generation of applications tailors health information to the individual user in real time and adapts tone, complexity and content to a person's stated circumstances and inferred profile. This mode of address is often called hyperindividualisation, and it departs from earlier forms of digital prevention in degree and arguably in kind. Where a printed leaflet or a static website offers the same material to everyone, a conversational system can in principle assemble a different message for each user on each occasion.

Personalisation of this sort is not new as an aspiration, and it rests on a substantial evidence base. Tailored health communication, in which message content is adapted to characteristics of the recipient, has been shown in meta-analytic work to outperform generic material for several behavioural outcomes (1). The promise of generative systems is that they extend this logic almost without limit, since the marginal cost of producing one further variant of a message falls towards zero for the provider.

The appeal of this development draws on a longstanding ambition of public health, namely the wish to reach groups that conventional prevention serves poorly. Vulnerability is the concept through which that ambition is usually expressed. Mechanic and Tanner describe vulnerability as a susceptibility to harm that arises from the interaction between the resources available to people and communities and the life challenges they face, and they locate its sources at developmental, personal, social and environmental levels (2). Frohlich and Potvin, examining vulnerable populations and the limits of the population approach, show that an intervention pitched at a whole population can widen rather than narrow health disparities when groups differ in their capacity to translate that intervention into health (3). Reaching the vulnerable has therefore never been a simple matter of delivery. Substance use is a particularly revealing case in which to pursue this question. Alcohol-related harm, for instance, falls disproportionately on socioeconomically disadvantaged groups, and it does so at given levels of consumption, once drinking patterns, smoking and body mass are accounted for (4). The inequality is therefore not explained by what disadvantaged drinkers do. The field thus offers a setting in which the consequences of hyperindividualisation for the concept of vulnerability can be seen with unusual clarity, and it frames the argument throughout.

It is tempting to see hyperindividualisation as the technical resolution of this old difficulty. If heterogeneity among the vulnerable is the obstacle, then a system that addresses each person individually appears to dissolve it. The present article questions that interpretation. The question it raises is not whether such systems work, but what happens to the concept of vulnerability itself once vulnerability becomes something that an algorithm can read and act upon. Geoffrey Rose's account of prevention gives the question its edge, since Rose showed that the relationship between individual risk and population health is neither simple nor intuitive (5).

The article introduces and develops a single proposition, which it calls the Vulnerability Transformation Hypothesis. Before stating it, the article distinguishes what is genuinely new in algorithmic hyperindividualisation from the older logic of tailoring on which it builds, since the force of the hypothesis depends on that distinction. The hypothesis itself is deliberately layered. It separates a modest and near-analytic claim about the level at which such systems act from a substantive claim about how they reconstruct vulnerability, and it keeps both apart from a contingent claim about the displacement of structural effort. The contribution is partly conceptual and partly empirical. It reframes what these systems do to the concept of vulnerability, and it offers predictions, one of which bears on the conceptual claim itself. The remainder of the article proceeds in steps. It reconstructs the public health understanding of vulnerability as a multilevel construct and recalls Rose's prevention paradox. It then describes how theory-driven behaviour change models are operationalised in digital systems, and it specifies how generative systems differ from earlier tailoring. On this basis it states the hypothesis, draws out a corollary concerning the prevention paradox, sets out four falsifiable predictions and illustrates the coding scheme on which they rely. It closes with the implications for prevention practice and for governance.

## 2 Vulnerability as a multilevel construct in public health

Vulnerability is among the organising concepts of public health, and its meaning is layered. In the account given by Mechanic and Tanner, vulnerability is not a fixed property of a person but an outcome of the interaction between resources and challenges, and it can arise at the level of the individual, the community or the wider population (2). On this view a single label covers developmental susceptibility, personal incapacity, disadvantaged social position, thin social networks and degraded environments, together with the way these factors accumulate and reinforce one another over the life course. The authors also note that different sources of vulnerability call for different kinds of response, ranging from individual medical care through to the social and economic development of neighbourhoods (2).

A second strand of the literature emphasises the relational and structural character of vulnerability. Bourgois and colleagues develop the concept of structural vulnerability to capture how hierarchies and power relations, rather than individual choices, expose certain groups to harm. They also propose an assessment covering eight domains, among them financial security, residence, social networks, legal status, education and discrimination (6). The purpose of their work is to shift attention upstream, away from the narrow range of risk behaviours that clinical and preventive encounters tend to record and towards the conditions that generate those behaviours in the first place (6). The theory of fundamental causes states this upstream emphasis in general form. Link and Phelan argue that social conditions persist as causes of ill health because they command resources that can be deployed against whatever risks happen to matter at a given time. Addressing a particular downstream mechanism therefore leaves the underlying cause able to act through others (7). On this account, the structural dimension is itself what reproduces individual risk over time.

Frohlich and Potvin connect this structural account to the design of interventions. Using the concept of vulnerable populations, they argue that population-level interventions may be translated into health gains more readily by those who are already advantaged, so that a measure intended to lift everyone can leave the relative position of disadvantaged groups unchanged or worse (3). Their argument matters here because it locates the difficulty of reaching the vulnerable not in the individuals who are hard to reach but in the social distribution of the capacity to benefit. Vulnerability, on this account, is a feature of social arrangements as much as of persons.

Substance use sharpens the conceptual question in ways that distinguish this field from many others in public health, because it shapes both how vulnerability is constituted and how prevention is received. For the substances most often at issue among young people, a degree of experimentation is widely regarded as ordinary rather than deviant. Parker and colleagues describe this through the normalisation thesis, which traces how certain forms of recreational substance use have moved from the margins towards the centre of youth culture. It identifies availability, rates of trying, rates of use, accommodating attitudes, especially among non-users, and a degree of cultural accommodation as its dimensions (8). Where a behaviour is normalised in this sense, abstention can even appear as the more unusual course. The vulnerability associated with the behaviour is then to some extent expected and accepted rather than treated as a failing. This normalised vulnerability sets substance use apart from risks that carry stigma from the outset, and it has consequences for the acceptability of intervention to which the article returns in Section 7.

## 3 The prevention paradox and the individualising pull of prevention

The modern case for population thinking in prevention was stated by Rose. He distinguished two questions that aetiology tends to conflate, the question of why a particular individual falls ill and the question of why one population has a higher incidence than another. He argued that the determinants of individual cases and the determinants of the incidence rate are not the same (5). From this distinction he drew the contrast between a high-risk strategy, which seeks out and protects susceptible individuals, and a population strategy, which seeks to shift the distribution of risk for everyone (5).

Rose also described what has come to be called the prevention paradox, the observation that a preventive measure bringing large benefits to a population may offer little to each participating individual (5). This paradox carries an important consequence for motivation and for acceptability. A measure that asks many people to change their behaviour in order to prevent comparatively few cases can seem unrewarding to the individuals who take part, and public support for it may be correspondingly weak (5). The high-risk strategy, by contrast, is intuitively attractive precisely because it concentrates attention on those who appear most likely to benefit.

It is this intuitive attraction that gives prevention what may be called an individualising pull. Resources, evaluation and political attention tend to flow towards interventions that identify and act upon individuals at elevated risk, since such interventions promise visible and attributable effects. In substance use prevention this pull favours advice directed at individual drinkers or smokers over measures acting on price, availability or marketing, although it is measures of the latter kind that act on the population-level conditions the prevention paradox brings into focus. The caution offered by Frohlich and Potvin applies with full force here, because the individualising pull can quietly widen the disparities that prevention is meant to reduce (3). The structural account of vulnerability and the population logic of Rose thus stand in tension with the individualising pull of practice. That tension is the backdrop against which algorithmic hyperindividualisation should be assessed.

## 4 Theory-driven behaviour change and its digital operationalisation

Applications that seek to change behaviour do not always rest on formal theory. Where they do, they commonly build on social-cognitive models, and the Health Action Process Approach is among the most widely used. The model, set out by Schwarzer, distinguishes a motivational phase in which an intention is formed from a volitional phase in which the intention is translated into action. It identifies risk perception, outcome expectancies and self-efficacy as the constructs that shape intention, with planning and further forms of self-efficacy supporting maintenance (9). The model is valued because it addresses the well-known gap between intention and behaviour, and it has been applied across many health domains (9).

The aim here is to describe this approach rather than to criticise it. The Health Action Process Approach is a careful and well-supported account of how individuals change health behaviour, and nothing in the present article depends on doubting its empirical standing. What matters for the argument is the level at which the model operates. Its constructs are attributes of persons, and its account of change runs through the cognitions and self-regulatory capacities of the individual. This suits the model's purpose. It is also the feature that becomes consequential once the model is combined with a technology capable of operationalising those constructs at scale.

Tailored communication is the mechanism through which such operationalisation has usually proceeded. By adapting messages to a recipient's standing on the relevant constructs, tailoring seeks to make health communication more relevant and therefore more effective, and meta-analytic evidence supports the value of this approach for several behaviours (1). A digital system that elicits a user's risk perception, confidence and circumstances, and that then selects or generates a message accordingly, is in effect operationalising a behaviour change model through tailoring. In substance use prevention this typically means eliciting a user's confidence about reducing their consumption, their perceived risks from drinking or smoking, and their stated triggers. The system then returns advice fitted to those individual states. Conversational agents built on this logic are an emerging presence in the field, with pilot randomised trials reported for alcohol reduction and for smoking cessation (10, 11). The next section asks what generative systems add to this established logic.

## 5 What is new in algorithmic hyperindividualisation

Hyperindividualisation can be described as tailoring carried to a new degree of granularity and adaptivity. Three steps make up the process. First, the system elicits signals from the user, whether through explicit self-report, through the content of what the user writes, or through the form and trajectory of the interaction. Second, it uses these signals to infer an evolving representation of the person, a structured profile expressed in terms of attributes and probabilities. Third, it generates or selects health information matched to that profile, and it can revise both the profile and the message continuously as the exchange proceeds.

Each of these steps operates on the individual. The signals are signals about a person, the profile is a profile of a person, and the generated message is addressed to a person. This follows from the design itself, since a system built to adapt its output to the user takes the user as its unit of analysis. The attributes such a system can represent are accordingly those that can be attached to an individual, among them risk perception, reported consumption, expressed confidence, language proficiency and sociodemographic markers.

The genuinely new must be separated from the old. The narrowing mechanism itself, the fact that the system can act only on the individual, is not new. It follows from the unit of analysis, and it is shared with printed leaflets and static websites. What generative systems add is of a different order. They infer attributes the user has not declared, reading them from the content and manner of the interaction itself. Inferred attributes can include mood, income bracket or life circumstances. Controlled work on large language models has shown that such inference is both accurate and cheap, recovering attributes such as location, income and sex from ordinary text at a small fraction of the cost of human annotation (12). They re-profile continuously, revising their representation of the person as the exchange unfolds, which carries into a conversational exchange the logic of just-in-time adaptive intervention that behavioural medicine has developed for mobile health (13). They generate rather than select, composing responses in open-ended natural language. And they operate at a scale where the marginal cost of one additional bespoke interaction approaches zero, orders of magnitude below the cost of a human-delivered equivalent (12).

These features do not all bear on the argument in the same way. Inference, scale and the opacity of the resulting profile deepen and extend the individual register, since they make the representation of the person finer, more comprehensive, harder for outsiders to inspect and cheaper to apply across a whole population. Continuous re-profiling adds temporal resolution but does not by itself change the level at which the system acts. Open-ended generation is the most interesting case, because it cuts both ways. A generative system could, in principle, identify a structural cause, telling a user that the availability of a substance in their area is the real problem and pointing them to a local initiative. This possibility matters, and the article returns to it in Sections 6.4 and 6.6. It does not disturb the central claim, because naming a structural cause in a message addressed to an individual is still an individual-level act. It informs a person; it does not change the availability. The novelty of these systems therefore lies in reach, autonomy and opacity, not in any new capacity to act on structure.

Hyperindividualisation is accordingly best understood as the upper reach of a continuum, the region in which inference, continuous re-profiling, open-ended generation and near-zero marginal cost combine. The predictions set out below treat depth of personalisation as a graded property precisely so that this boundary need not be drawn arbitrarily.

This is the point at which the concept of vulnerability enters the machinery. If a prevention system is to reach the vulnerable more precisely, it must first render vulnerability in a form it can process, which is to say as a set of individual attributes. The next section addresses what becomes of vulnerability once it has been rendered in this way. In particular, it asks whether the structural and relational dimensions described earlier can survive a translation that operates entirely at the level of the individual.

## 6 The vulnerability transformation hypothesis

### 6.1 The claim in brief

The central thesis of this article can be stated briefly. In substance use prevention, algorithmic hyperindividualisation tends to reconstruct the operationalised concept of vulnerability from a structural and relational construct into a constellation of individual attributes. The claim is conditional, and it holds for any system that presents itself as a means of reaching the vulnerable. For such a system, the more precisely it stratifies individual risk, the smaller the part of vulnerability it can act upon. In a system addressing alcohol or tobacco use, for instance, a person's local availability of these substances or the prevailing norms of their neighbourhood can enter only as attributes attached to the person, never as conditions the message itself can alter. In short, individual precision and structural narrowing are one operation seen from two sides. The claim is meant to be falsifiable, and the predictions set out below say what would count against it. The remainder of this section first separates the three component claims of the thesis and gives them names. It then explains why re-encoding differs from mere omission. It locates what is unavoidable at the level of the device and what remains open at the level of the field, and it closes with an empirical anchor.

### 6.2 The three component claims

The thesis combines three claims of decreasing certainty, and it helps to name them.

The first may be called the *addressability claim*, and it is almost analytic. A system that represents and addresses users one by one has only individual-level levers, simply by virtue of what it is. Taken alone, this claim is modest, and it is not yet the thesis.

The second, the *re-encoding claim*, is the substantive one, and it is conditional. For any system that presents itself as a means of reaching the vulnerable, the operationalised concept of vulnerability is reconstructed into individual attributes, and its structural and relational dimensions fall outside what the system can act upon. A user who lives in a deprived neighbourhood, to introduce an example that will recur, enters such a system as a person with a particular risk profile, and the deprivation enters with them, but only as a feature of that profile. Where a system makes no claim about vulnerability, the structural dimension is merely absent rather than transformed, and the point is correspondingly weaker. Whether and how often such systems invoke vulnerability or the cognate goal of reaching underserved groups is itself an empirical question, one that the predictions below are designed to test rather than assume. The substance use applications used here for illustration are a case in point in both directions. Some present themselves in the language of reaching at-risk or underserved young people, while others are framed as harm reduction or informed choice. The claim bites for the former and remains silent on the latter.

The third, the *displacement claim*, is contingent. At the level of the field rather than the single system, the visible success of individualised tools may draw attention and resources away from structural measures. The article does not rest on this claim; it returns to the evidence for it in Section 9. Figure 1 sets out the three claims, their differing degrees of certainty, and the level at which each operates.

**Figure 1:**
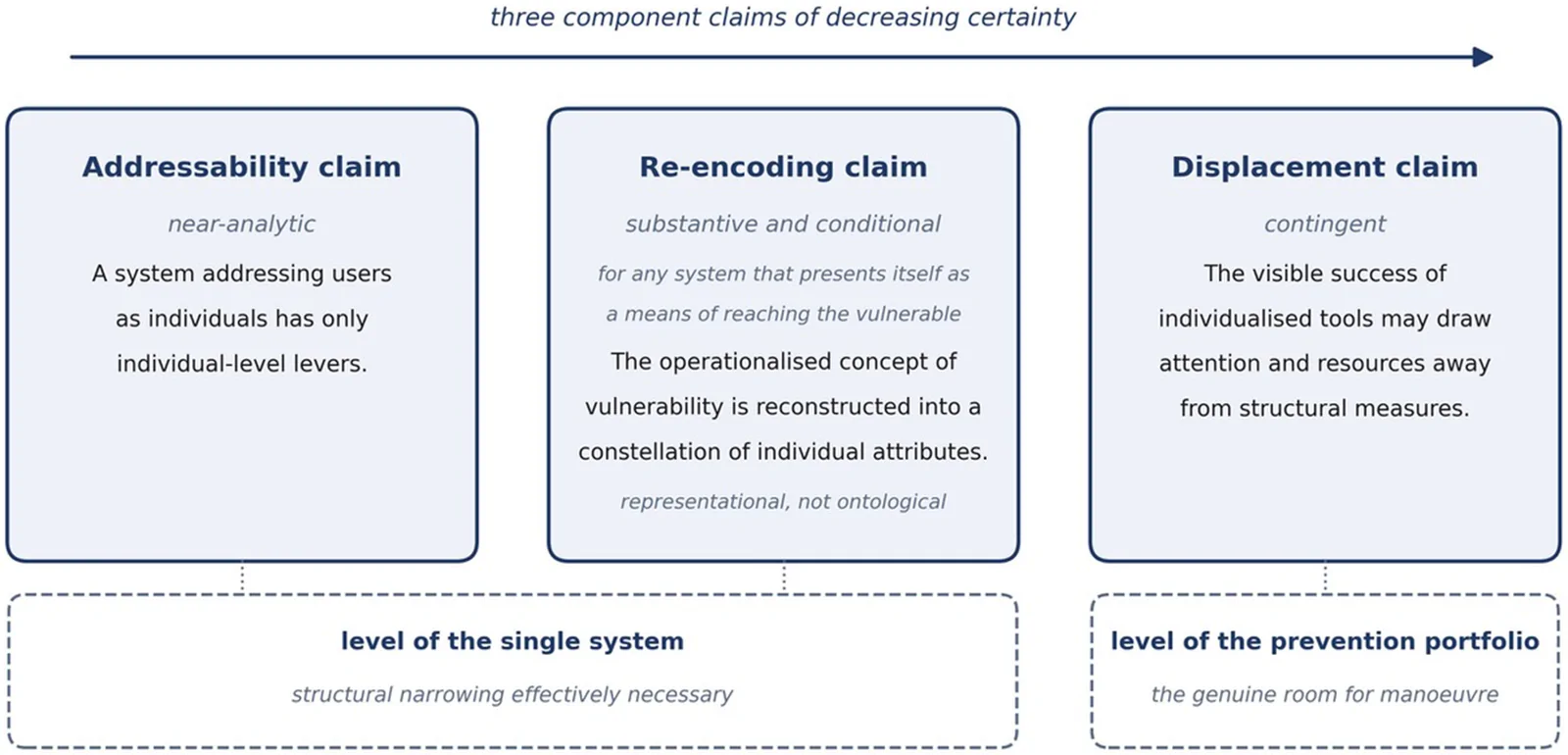
The architecture of the vulnerability transformation hypothesis. The hypothesis combines three component claims of decreasing certainty. The addressability claim and the conditional re-encoding claim concern the single system, where the structural narrowing is effectively necessary. The displacement claim concerns the prevention portfolio, where the genuine room for manoeuvre lies. The transformation described by the re-encoding claim is representational rather than ontological.

### 6.3 Why re-encoding is not omission

The re-encoding claim rests on a point about representation, best seen through an obvious objection. One might suppose that a sufficiently rich system could ingest structural information, for instance a user's neighbourhood, income or educational background, and thereby retain the structural dimension. It cannot, and the reason is instructive. When structural information is entered into an individual risk model, it does not re-enter as structure. It re-enters as an attribute of the individual, a predictor that sharpens the estimate of this person's risk. The information is not lost; its role changes. Section 8 below formalises this role as context rather than target. Consider again the user from the deprived neighbourhood. The system may learn where they live, and it may tailor its message accordingly, yet it continues to address the user rather than the deprivation. From where they live, it may infer something about the local availability of alcohol or tobacco and adjust its advice accordingly. The availability itself remains exactly as it was. The structural condition becomes a feature of the person, and the available lever remains an individual one. This is why the result is a narrowing rather than a neutral change of medium. Figure 2 shows this pathway, together with the pathway the system does not have.

**Figure 2:**
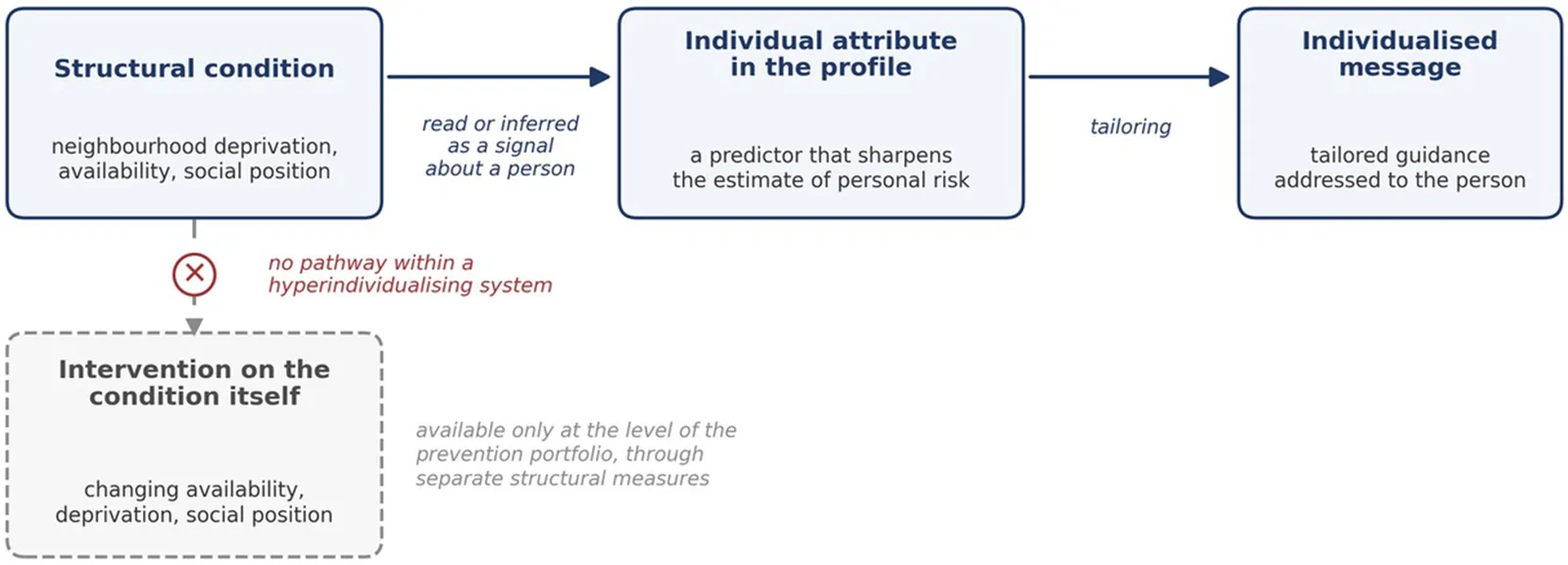
The re-encoding pathway at the centre of the vulnerability transformation hypothesis. Structural information enters a hyperindividualising system as a signal about a person, is represented as an individual attribute that sharpens the estimate of that person’s risk, and shapes a message addressed to the individual. A pathway from the represented condition to an intervention on the condition itself is not available within the system. At the level of the prevention portfolio, it must be supplied by separate structural measures.

It is the conditional premise that justifies speaking of a *transformation* at all. Two qualifications make the word exact. The first concerns the premise. These systems are commonly presented and justified as means of reaching the vulnerable, which is to say as operationalisations of vulnerability itself, and the argument bites precisely there and not where a system makes no such claim. The second concerns the kind of transformation at issue. When a construct defined at several levels is operationalised by a mechanism that can represent only one of them, and the resulting individual-level proxy is carried forward under the original name, the concept that does the work in practice is no longer the concept that was named. The label is retained while its referent is quietly exchanged. This is a transformation of the operationalised concept, not of the world it describes. The structural causes do not dissolve when a system ceases to represent them. As the theory of fundamental causes insists, they persist and continue to act. What changes is what prevention, so equipped, takes vulnerability to be and can address. The transformation is therefore representational rather than ontological, and the article's claim should be read in that sense throughout.

### 6.4 What is unavoidable and what remains open

The theory of fundamental causes also explains why, at the level of the single system, this narrowing is effectively unavoidable. As Section 2 set out, a fundamental cause commands resources that can be redeployed against whatever risks are salient. Addressing a single downstream mechanism still leaves it other pathways through which to act (7). An intervention pitched at the individual acts on proximal mechanisms and cannot reach a fundamental cause. A hyperindividualising system is such an intervention, and, as the previous section argued, even its most open-ended output can name a structural condition but cannot act on one. At the level of the system, therefore, the structural narrowing is effectively necessary. The genuine room for manoeuvre lies at the level of the prevention portfolio, where such a system can be embedded among structural measures that it does not itself contain. This is why the contingency belongs to the displacement claim rather than the re-encoding claim. The limitation is in any case shared with any individually pitched measure and is not peculiar to artificial intelligence. What is peculiar to these systems is that inference, near-zero marginal cost and the opacity of the profile give the limitation an unusual reach and an unusual capacity to pass unnoticed. The narrowing lies in the system's design rather than in any single message. Aggregate behavioural change can alter environments over time, and a system may name a structural cause and refer a user to an initiative that acts collectively. Section 6.6 sets out why such pathways, which run through actors outside the system, mark the boundary of the claim rather than a counter-example to it.

### 6.5 An empirical anchor and the assembled claim

The work of Obermeyer and colleagues both illustrates the pattern and shows precisely where its correction stops. A widely used clinical algorithm predicted health needs from healthcare expenditure, and because less is spent on equally sick patients from disadvantaged groups, it underestimated those patients' needs, reproducing a structural inequality as a difference in individual risk (14). The distortion arose through exactly the mechanism the re-encoding claim describes, a structural fact entering the model as an individual signal, and it proved largely correctable once recognised, by re-specifying the outcome the model was built to predict (14). Here it is easy to conflate two things. The correction required attention to a structural distortion, which is to say a deliberate act rather than a better fit to the original target. But what the correction produced was a better individual measure, a truer estimate of individual health need, not an instrument that acts on the unequal spending that caused the distortion. De-biasing removed a misrepresentation; it did not convert an individual tool into a structural one. Obermeyer therefore supports the careful claim twice over, since it shows that the default encodes structure as individual risk, and that even a fully corrected system remains individual in its reach. The case also tempers a one-sided interpretation of the critical literature. Eubanks, O'Neil and Benjamin document how data-driven systems encode and conceal social hierarchy (15–17). Yet the corrected algorithm is a reminder that such systems can at least be made to expose inequality, even where they cannot undo it. The same structure is visible in substance use prevention. A system that reads a person's neighbourhood as a predictor and tailors its advice more finely has improved an individual estimate, not touched the availability of alcohol or tobacco or the social conditions that the structural account treats as fundamental.

Read together, these considerations yield the claim of this article. What a hyperindividualising system can do about vulnerability is bounded by what it can represent, and what it can represent are individual attributes. Structural conditions enter only by being recoded as such attributes, at which point they function as predictors of individual risk rather than as objects that might be changed. The finer the individual discrimination, the more completely the operationalised concept of vulnerability is rendered in individual terms. At the level of the device this is effectively unavoidable; at the level of the field it can be offset only by pursuing structural conditions through other means, deliberately and separately. Precision and structural narrowing are, on this account, two descriptions of one operation, and the structural causes they leave untouched do not thereby disappear.

To forestall misreading, the thesis does not hold that personalisation is ineffective, that the use of artificial intelligence in prevention is illegitimate, or that the narrowing cannot be offset. It holds that such systems act on some dimensions of vulnerability and not others, and that their success in the individual register tends to obscure the dimensions they cannot reach. Offsetting this requires making structural conditions an explicit target, which these systems do not do on their own.

### 6.6 Boundary conditions and what would count against the hypothesis

The three claims concern how a system represents the problem it addresses and what it can take as a target. They do not concern every consequence that may follow from its use. This distinction marks the boundary of the hypothesis, and two objections turn on it.

The first objection is that individual change may alter structural conditions over time. If enough users reduce their consumption, local norms may shift, and demand may fall in ways that eventually bear on availability. The second is that a system may name a structural cause and refer a user to a community initiative, which may in turn contribute to collective action and to policy change. Both observations are correct, and neither bears on the claims. In each case, actors and processes outside the system produce the structural effect and reach the structural level only through them. The system does not represent that pathway, cannot condition its action on it, and does not measure its success by it. What it represents remains a person, and what it can address remains that person's attributes. The claim concerns the model of the problem the system carries, not the totality of what may follow from its use.

Stating the boundary in this way makes clear what would count against the hypothesis. The addressability claim would fail if a system that addresses users individually could itself alter a structural condition, for instance by acting on the licensing, pricing or physical availability of a substance rather than by prompting a user to act. The re-encoding claim, and with it the first prediction, would fail if work that invokes vulnerability were found to operationalise it structurally. A digital tool that supports licensing authorities in mapping outlet density, presented as a means of protecting vulnerable communities, would be such a case, since it invokes vulnerability and targets availability rather than a person. A weaker case would be a hyperindividualising system that invokes vulnerability, tailors to individual attributes, and states that the structural dimension lies outside its reach and must be addressed by other measures. Such a system does not present the individual proxy as the means of reaching the vulnerable, and the concept is left intact. The displacement claim would fail if depth of personalisation and structural share were found to move together, or not to move at all, within bands of comparable intervention type. None of these outcomes is excluded by the definitions, and each is a matter for empirical appraisal rather than argument.

## 7 The algorithmic prevention paradox and the question of acceptability

A corollary follows when the hypothesis is set beside Rose's account. Rose's paradox held that a population measure may bring little apparent benefit to each individual who takes part in it (5). Hyperindividualisation appears to invert this picture, since it offers each user a visibly tailored and personally relevant intervention. The inversion is only apparent. By concentrating the entire preventive effort in the individual register, the hyperindividualising system maximises individual relevance while leaving the determinants of the incidence rate untouched. The more successfully it speaks to each person, the further it travels from the population causes that Rose argued should be the prior concern (5). This may be called the algorithmic prevention paradox. It is the situation in which an intervention becomes ever more precisely targeted at individuals while its capacity to alter the structural causes of incidence diminishes.

A second and more tentative observation concerns acceptability, which bears on whether such systems will be adopted rather than on the conceptual claim that is the article's main concern. Here a distinction between two populations must be kept in view, because the substance field contains both and they are too readily conflated. Problematic or dependent use attracts heavy stigma. Room shows that dependence, and the loss of control the term names, attract near-universal moralisation and stigma, and that this stigma is itself entangled with social inequality (18). Recreational use among young people occupies a different position. The normalisation thesis describes it as an ordinary feature of youth culture, and its subjects are, on the whole, not stigmatised in the way that dependent users are (8). These are distinct groups in distinct moral positions, and a single statement about acceptability cannot cover both.

The distinction matters for prevention of the kind at issue here, which addresses young people who use or might use recreational substances rather than those with a diagnosed dependence. For this population, the salient pressure is less stigma than normalisation, since an intensively personalised intervention aimed at a behaviour widely regarded as ordinary may be experienced as disproportionate or unwelcome by its recipients (8). Stigma enters at a different level, that of the political economy of the field as a whole. Room documents the moralisation of dependence (18), and a conspicuous and costly investment in a problem seen in that light may attract resentment. Neither pressure is dissolved by greater personalisation, and neither belongs to the conceptual core of the hypothesis.

This raises a question the hypothesis must face directly. If the relevant group is composed of young people for whom recreational use is normalised, and who are not a conspicuously stigmatised or marginalised population, in what sense are they vulnerable at all, and is the vulnerability lens even apt here? The apparent tension dissolves on closer inspection. Seeing why strengthens rather than weakens the argument. The vulnerability at issue in a normalised behaviour is not concentrated in a visibly disadvantaged few but distributed across a population. It is patterned by exactly the structural conditions the hypothesis foregrounds, namely the availability of substances, prevailing norms, and the educational and economic circumstances that shape who is exposed and who is harmed. This is vulnerability in Rose's population sense (5) rather than in the sense of a high-risk minority. Precisely because it is diffuse and structurally patterned, an apparatus that renders it as the risk profile of each separate individual is especially ill-suited to it. The re-encoding that the hypothesis describes is, for this group, not a marginal distortion but the central one.

## 8 Testability and empirical predictions

### 8.1 Two roles for structural factors, and an inventory for coding

A hypothesis of this kind earns its place only if it can be tested, and the present one can be specified as a set of predictions about how vulnerability is treated in research and practice. To state the predictions precisely, a distinction is needed between two ways in which a structural factor can appear in a prevention system. A structural factor may appear as context, that is, as a predictor that sharpens an estimate of an individual's risk, or it may appear as a target, that is, as a condition that the intervention sets out to change and measures as an outcome. The hypothesis concerns the balance between these two roles, and the predictions below are framed so that evidence could count against them.

An inventory of structural dimensions is needed to ensure consistent coding. The PROGRESS framework offers a suitable and widely used inventory, since it lists place of residence, race or ethnicity, occupation, gender, religion, education, socioeconomic status and social capital as factors that stratify health opportunities and outcomes (19). For each prevention system or study, one can record whether each of these dimensions is absent, present as context, or present as a target. In parallel, one can record the depth of personalisation, understood as the number and granularity of individual attributes the system uses to adapt its output. Two clarifications keep the coding precise. The dimensions of this inventory are often recorded as attributes of persons, so the inventory marks where social stratification arises rather than supplying a ready-made structural ontology. What the coding turns on is the role a dimension plays, whether it is engaged as a modifiable social condition, which counts as a target, or as a person-level stratifier that adjusts an individual estimate, which counts as context. In substance use prevention, place of residence stands in for the local availability of alcohol and tobacco and for neighbourhood norms, among other features of the place. The question for coding is whether a system treats such a factor as a modifiable condition or merely as a sharpening of an individual estimate. Applied to real systems, the inventory needs a procedure as well as a scheme. Each system should be coded independently by two researchers against a codebook that fixes, for every dimension, what counts as a target and what counts as context, with anchor examples drawn from documented systems. Agreement should be reported with a chance-corrected statistic, and disagreements resolved with a third coder, with the rate and location of disagreement reported rather than absorbed. Real systems are messier than the contrast in Section 8.3 suggests, and the dimensions on which coders diverge are themselves informative, since they mark where the distinction between context and target is genuinely contested.

### 8.2 Four predictions

There are four predictions, and the first is included precisely because it bears on the conceptual claim that the others do not. The first, discursive, prediction concerns systems and studies that invoke vulnerability, equity, or the goal of reaching underserved or hard-to-reach groups. The prediction is twofold. When its methods are examined, such work will operationalise vulnerability through individual-level attributes rather than through the structural dimensions of the inventory. In the stronger and more telling part, the individual proxy will be presented as the very means by which the invoked vulnerability is addressed. The system or study then treats reaching the vulnerable and tailoring to individual attributes as one and the same. It is this presented identification, and not the mere co-occurrence of vulnerability language with methods aimed at individuals, that would evidence a substitution of the concept rather than simple inconsistency of exposition. Work that invoked vulnerability and then pursued structural targets, or that kept the structural construct distinct from its individual measures, would count against the hypothesis. Such work is possible, which is what keeps the prediction contingent rather than true by construction. Section 6.6 sets out two forms it could take.

The second and third predictions concern practice and are, by comparison, confirmatory, since they fix in detail the baseline against which the others are read. The second is that, across research and applications in digital and artificial-intelligence-supported substance use prevention, vulnerability is operationalised predominantly through individual-level attributes such as risk perception, consumption patterns and sociodemographic markers. The third is that, where structural dimensions do appear, they appear overwhelmingly as context, that is, as inputs that inform the estimate of individual risk without being set as targets of intervention or measured as outcomes.

The fourth prediction concerns the coupling at the centre of the hypothesis, that depth of personalisation and the structural share move in opposite directions, so that more deeply personalised systems address a smaller share of structural dimensions as targets. Two cautions govern its test. First, if it were tested only on hyperindividualised systems, it would fail for want of variance, since these cluster at one end of both dimensions, personalisation depth and structural share. The test of the fourth prediction must span the spectrum of personalisation, from population-level and setting-based digital interventions through moderately tailored programmes to hyperindividualised systems. Second, and less obviously, a negative association observed only across intervention types would be close to trivial, because setting-based interventions address settings, and individual ones address individuals, almost by definition. The prediction is therefore staked on the stronger and riskier version, that the structural share continues to decline with personalisation depth within bands of comparable intervention type, or once intervention type is held constant. It is this within-type association, beyond what the type labels already imply, that would genuinely corroborate the hypothesis, and the absence that would tell against it. The measurement is demanding and is itself part of what a test would establish, since depth of personalisation and structural share are assessed on different scales and must be rendered comparable, for instance as ordered categories or as proportions defined per intervention. Table 1 summarises the four predictions, together with the evidence that would corroborate each and the evidence that would tell against it.

**Table 1:** The four predictions of the vulnerability transformation hypothesis, with the evidence that would corroborate each prediction and the evidence that would tell against it.

Prediction	Evidence that would corroborate	Evidence that would tell against
**P1 (discursive):** Work that invokes vulnerability, equity or the goal of reaching underserved groups operationalises it through individual-level attributes and presents the individual proxy as the very means by which the invoked vulnerability is addressed.	Framings that name vulnerability while methods measure only individual attributes, with tailoring presented as the very means by which the vulnerable are reached.	Work that invokes vulnerability and pursues structural targets, or keeps the structural construct distinct from its individual measures.
**P2 (operationalisation):** Across digital and AI-supported substance use prevention, vulnerability is operationalised predominantly through individual-level attributes.	Dominance of risk perception, consumption patterns and sociodemographic markers in how vulnerability is measured.	A substantial share of work operationalising vulnerability through the structural dimensions of the inventory.
**P3 (context, not target):** Where structural dimensions appear, they appear overwhelmingly as context rather than as targets.	Structural dimensions used as predictors that sharpen individual risk estimates, without being intervened on or measured as outcomes.	Structural dimensions regularly set as objects of intervention and measured as outcomes.
**P4 (coupling):** Depth of personalisation and the structural share move in opposite directions, within bands of comparable intervention type or once type is held constant.	A negative within-type association between personalisation depth and the share of structural dimensions addressed as targets.	No within-type association, or a positive one, once intervention type is held constant.

### 8.3 An illustration and the appropriate test

A brief illustration on documented systems shows that the coding scheme discriminates rather than merely registering the answer it was built to give. Consider two cases. The first is QuitBot, a quit-smoking programme delivered as a conversational chatbot and evaluated in a pilot randomised trial, which combines a structured sequence of short daily conversations with a large-language-model function that answers users' open-ended questions (11). Its content covers motivation, quit dates, cessation medication, coping with triggers, and recovery from lapses. Coded against the inventory, it engages place of residence only as a contextual input that localises its advice, treats education, occupation and socioeconomic status, where these enter, as predictors that adjust the message, and uses gender and age as personalisation parameters. No dimension appears as a target, because the system has no instrument that could act on any of them. The second case was chosen because its surface features suggested a collective object, so that it could have cut against the expected pattern. Group-delivered digital interventions in chronic disease self-management, such as instruction through a moderated messaging group or a social-media microlearning format, are conducted in a shared channel (20, 21). Coded against the inventory they nonetheless come out individual. The group is the medium of delivery, while the targets are the knowledge, self-efficacy and self-care of each participant, and the outcomes are measured accordingly at the individual level. The scheme therefore separates the channel through which an intervention reaches people from the level at which it acts, and it does so against the surface expectation rather than in line with it. The second case also shows how sparse the structural end of the spectrum is. The most clearly structural levers, such as the regulation of availability, lie largely outside what any digital intervention can target, and a candidate that looked collective proved on inspection to be individual. Section 6.6 describes what a genuinely structural case would look like. That sparseness is consistent with the hypothesis, and documenting it is part of what a systematic appraisal would have to do. The exercise is illustrative rather than empirical.

A systematic mapping of how vulnerability is conceptualised, measured and acted upon across this literature and the available systems would provide the appropriate test. The discursive and the coupling predictions deserve the closest attention, the first because it bears on the conceptual claim and the second because it concerns a relationship between measures rather than either alone. A finding in either direction would be informative for the field. For the coupling prediction, a feasible design would draw a sampling frame from trial registries and application repositories and restrict comparison to bands of comparable intervention type. Each system would then be coded for depth of personalisation and for the share of structural dimensions treated as targets, and the association would be examined within those bands. Repeating the coding across successive versions of the same systems would allow the prediction to be tested over time as well as across systems.

## 9 Implications

If the hypothesis holds, the first implication concerns the balance between behavioural and structural prevention, and it is here that the contingent displacement claim becomes important. Hyperindividualisation is a powerful instrument of behavioural prevention, and its arrival may shift attention and resources further towards the individual register at the expense of measures that act on conditions. At the level of the field, this is a documented tendency rather than a necessity. Critical scholarship has given this pattern the name of lifestyle drift, describing how stated commitments to the social determinants of health give way, in implementation, to programmes aimed at changing what individuals do (22). A technology that excels at the individual level and can point to visible individual successes is well suited to accelerate that drift. The risk is not that personalised systems do harm in themselves, but that their visible success crowds out the less tractable work of changing the determinants of incidence. Preserving the balance that Rose and that Frohlich and Potvin commend requires that structural vulnerability remain an explicit object of prevention even as individual tools become more capable (3, 5).

Two neighbouring debates help to place the contribution. The first is lifestyle drift, which describes how policy repeatedly relocates upstream causes downstream as individual behaviour (22). The second is the critique of precision public health, which warns that the field is at risk of becoming precision medicine at population scale, and that unless social position is kept central as a determinant of health it will not reach the causes of health inequities (23). The present article shares the concerns of both, but its claim is narrower and more specific than either. It is not only that responsibility is shifted, as in lifestyle drift, nor only that precision may fail to reach the causes of inequity, as in the precision-public-health critique. It is that the operationalised concept of vulnerability itself is re-encoded, its referent exchanged while its name is kept, by a particular class of systems and in virtue of how they represent the people they address. That is the contribution the hypothesis offers to these neighbouring discussions.

A second implication concerns governance. The reconstruction of vulnerability as individual data raises questions about the data on which such systems are built, about transparency in how risk is inferred, and about accountability for the populations a system may quietly underserve. The tendency of data-driven systems to reproduce inequality while appearing neutral is directly relevant to these questions (14–17). However, there is a countervailing possibility, illustrated by the correction of the algorithm discussed earlier, that such systems can be made to surface and reduce inequality when that is set as an explicit aim (14). Regulatory frameworks for artificial intelligence and for the use of health data are the arena in which these questions will be settled. They should attend not only to the accuracy and safety of individual predictions but also to whether structural conditions are anywhere made a target. It is the absence of structural targeting, rather than any defect of individual prediction, that the hypothesis identifies as the danger.

A third implication is conceptual. If the field adopts hyperindividualisation without examining what it does to the meaning of vulnerability, it risks narrowing that meaning by default, so that vulnerability comes to denote whatever an algorithm can measure. Keeping the multilevel and structural understanding of vulnerability in view is therefore a matter of conceptual care as much as of intervention design, and it is a task for the research community as much as for developers and regulators.

A final remark concerns the scope of the hypothesis. Nothing in the three claims is specific to substances, and the mechanism should operate wherever a hyperindividualising system presents itself as a means of reaching the vulnerable. Substance use prevention is nonetheless the field in which the mechanism is most clearly visible, and three features distinguish it from other prevention fields. Reaching the vulnerable is an explicit part of how programmes here describe and justify themselves, more than in most other areas of prevention, so the condition of the re-encoding claim is met by design rather than by accident. The structural levers are unusually well specified and well evidenced, from price and availability to marketing, so the distance between what a system can target and what the field takes to be decisive is easy to see. And the normalisation of use described in Section 7 makes the vulnerability concept unusually elastic, which is the condition under which its quiet substitution is most likely to pass unremarked. Chronic disease self-management offers the instructive contrast. Digital interventions for diabetes and cardiovascular risk tailor to individual attributes in much the same way, and the group-delivered formats discussed in Section 8.3 turn out to be individual in their targets despite their collective channel (20, 21). The vulnerability vocabulary is less central to how those fields justify themselves, however, and their structural levers, from the food environment to the built environment, are more diffuse than price and availability. The same re-encoding should occur there, and it should be harder to detect. That is a claim about where the mechanism will be visible rather than a restriction of its scope, and it can be tested with the scheme of Section 8 unchanged.

## 10 Conclusion

This article has proposed the Vulnerability Transformation Hypothesis. For systems presenting themselves as means of reaching the vulnerable, algorithmic hyperindividualisation in substance use prevention reconstructs the operationalised concept of vulnerability from a structural and relational construct into a constellation of individual attributes, so that gains in individual precision and the narrowing of structural reach move together. The argument has distinguished what is new in these systems from the tailoring they extend. It has separated the near-analytic addressability claim from the substantive and conditional re-encoding claim, and both from the contingent displacement claim. It has specified that the transformation is representational rather than ontological, since the structural causes persist while receding from what such systems represent and can change. At the level of the single device, the narrowing is effectively unavoidable; the room to act lies at the level of the field. The argument has drawn on the public health understanding of vulnerability as a multilevel construct, the theory of fundamental causes, Rose's account of the prevention paradox, and critical scholarship concerning algorithmic systems and precision public health.

The hypothesis is stated so that it could be wrong, and the predictions and coding scheme show how. One prediction is discursive and brings even the conceptual claim within reach of evidence. Whether the coupling proves as general as claimed is an empirical matter, and whether the displacement it warns of occurs is more empirical still. The article will have served its purpose if it equips the research community to find out, and if it keeps the structural dimension of vulnerability in view while the individual tools grow more capable.

## Data Availability

The original contributions presented in the study are included in the article; further inquiries can be directed to the corresponding author.
